# Social capital and diabetes care: a cross-country analysis of prevalence, diagnosis and glycaemic control in 11 European countries using SHARE data

**DOI:** 10.1186/s12889-026-27352-x

**Published:** 2026-05-25

**Authors:** Laura Ceurvels, Jorik Vergauwen, Edwin Wouters

**Affiliations:** Department of Sociology, University of Antwerp, Sint-Jacobstraat 2, Antwerp, 2000 Belgium

**Keywords:** Diabetes, Social capital, Cascade of Care, Prevalence, Diagnosis, Control

## Abstract

**Background:**

Diabetes is a major health issue that is increasingly prevalent in Europe. Providing high-quality care is crucial, as failing to do so can lead to various complications. This study employed a cascade-of-care approach and data from 11 European countries to identify the points at which patients disengage from the diabetes care continuum, and explored the relationship with social capital variables, sociodemographic factors, and health indicators.

**Methods:**

Using cross-country data from Wave 6 and its related dried blood spot sample of the Survey of Health, Ageing and Retirement in Europe, multilevel logistic regression models were constructed using a stepwise approach. The stages of the cascade of care were outcome variables: prevalence of diabetes, receiving a diabetes diagnosis, and achieving glycaemic control based on HbA1c levels.

**Results:**

Higher levels of bridging social capital are associated with lower diabetes prevalence, partly mediated by sociodemographic factors and health indicators. Diabetes is more commonly diagnosed in older individuals (although this link weakens in the oldest age groups), men, and those in poorer health. Better HbA1c control is associated with having a partner in the household, lower BMI, and being a woman.

**Conclusion:**

This study highlights that social capital is associated with diabetes care among older adults in Europe. Bridging social capital can reduce prevalence, and having a partner can enhance diabetes control. Social capital also appears to be indirectly related to receiving a diagnosis. This reinforces the need for a combined medical and social approach to diabetes care.

## Background

 Diabetes is a severe health issue that is becoming increasingly prevalent in Europe. It currently affects around 64 million adults [1], while projections indicate a further 10% increase in cases between 2024 and 2050 [2]. Providing high-quality care is crucial since failing to follow prescribed treatments or maintain a healthy lifestyle can result in complications. These may include damage to blood vessels in the heart, eyes, kidneys and nerves, which can cause vision problems, kidney dysfunction and issues affecting the limbs [1, 3].

Rather than technical healthcare challenges, diabetes care is often characterised by organisational complexity [4]. Effective diabetes management requires ongoing behavioural change and adherence to treatment protocols and medication. This necessitates coordinated action from various stakeholders, including the healthcare system, doctors, patients, and their support networks [4, 5]. This complexity can be captured by the care cascade, a model outlining the steps involved in managing the condition [6, 7]. The steps in this model include the prevalence of diabetes, receiving a diagnosis, and controlling the disease. Individuals may become disengaged at any stage of this process [6–9]. In a cross-country study of 74 countries, Beagley [9] found that 45.8% of diabetes cases remained undiagnosed, with 83.8% of these occurring in low- and middle-income countries, yet countries with advanced healthcare systems also faced difficulties. In the UK, over 35% of diabetes patients did not reach the recommended HbA1c target, and among 15 European countries, diabetes prevalence ranged from 5.1% to 16.5%, with 29.3% to 36.6% of these cases undiagnosed [9]. Data from the international diabetes federation (IDF) and the world health organization (WHO) confirm that about one-third of European diabetes cases are undiagnosed, and up to half are not meeting treatment targets [2, 10].

Disparities in the care cascade are not random but are shaped by inequalities associated with social and economic factors [3, 11]. Whilst most preceding studies have concentrated on socio-economic determinants [12, 13], comparatively less attention has been paid to the role of social factors. Successfully managing a chronic disease such as diabetes requires patients to navigate complex healthcare systems, adhere to treatment plans, and sustain long-term behavioural changes. These responsibilities are partly shaped by social relationships, influencing lifestyle choices, access to care and information, and the level of available support [14–17].

The concept of social capital, which is a social determinant of health, captures these social relationships and the resources they offer. It encompasses the value gained from social networks, trust, and shared norms that foster cooperation and support [18–20], thereby impacting a person’s health [21, 22]. According to Putnam’s work Bowling Alone [19], social capital can be divided into two types: bonding and bridging. Bonding involves close-knit ties offering emotional and practical support, while bridging connects diverse groups, providing access to new information and resources.

Previous research into the relationship between social capital and diabetes has typically focused on either the prevalence or the control over the condition. While studies generally agree on the protective effect of social capital against diabetes prevalence [23–25], the results regarding the relationship between social capital and diabetes control have been more ambiguous [21, 26–28]. For example, higher levels of social capital are associated with lower levels of depression and greater physical activity among people with diabetes [27]. Living in more collaborative neighbourhoods is related to better glucose control [26], and stronger community trust and a greater sense of control over institutions correspond to improved diabetes management [28]. However, these studies find no significant associations between social capital and glycaemic control [27], or between glycaemic control and factors such as participation in local organisations, the overall social capital index, or neighbourhood trust [26].

This study aims to address four gaps in the existing literature. First, while recent research increasingly investigates the link between social capital and diabetes, most studies focus on either prevalence or management. This study considers multiple stages of the diabetes care cascade to provide a more comprehensive view of the care continuum. Second, by examining both bonding and bridging social capital, our analysis offers a more comprehensive understanding of how these forms of ties relate to diabetes care. Third, most previous studies rely on general or subjective health measures, such as self-rated health. In contrast, this research utilises biomarkers to assess glycaemic values, providing more objective evidence [21, 29]. Fourth, while previous research on the care cascade has often focused on single countries or combined disparate data sources, this study employs harmonised, cross-national survey data from the SHARE project, ensuring greater consistency and reliability.

This research aims to offer further insight into the relationship between social capital and diabetes care. Drawing on Putnam’s [19] distinction, we hypothesise that bonding and bridging social capital are associated with different stages of diabetes care. Bonding social capital may promote healthy behaviours, encourage help-seeking, and support disease management. Therefore, the study expects that a higher level of bonding social capital corresponds to lower diabetes prevalence (Hypothesis 1a), increased likelihood of diagnosis (Hypothesis 1b), and improved diabetes control (Hypothesis 1c). Additionally, bridging social capital facilitates the dissemination of health-related information and improves access to resources. Accordingly, the study posits that higher levels of bridging social capital are associated with lower diabetes prevalence (Hypothesis 2a), a higher likelihood of diagnosis (Hypothesis 2b), and better diabetes control (Hypothesis 2c).

## Data and methods

### Data

The study used data from the Survey of Health, Ageing, and Retirement in Europe (SHARE) [30, 31], a comprehensive panel survey of adults aged 50 + and their cohabiting partners. To address our research questions, we analysed data from SHARE Wave 6 (2015) [32] and its related dried blood spot (DBS) samples [33–35]. Approximately 27 200 participants from twelve countries consented to DBS collection, yielding a 67% participation rate among eligible respondents. Of these, 22 598 (83.1%) provided at least one blood parameter, and 19 785 (72.8%) specifically had data on the diabetes marker, HbA1c^1^. Israel was excluded due to over 50% missing HbA1c data. After this exclusion, 19 176 (70.5%) respondents with diabetes measurements remained. An additional 2 183 (11.4%) respondents were excluded due to missing values for at least one key variable, resulting in 16 993 complete cases for analysis (62.5% of the original 27,200 who consented). All methods and data collection practices were performed in accordance with the relevant guidelines and regulations (Declarations of Helsinki).

#### Constructing the diabetes care cascade

To construct the diabetes care cascade, we distinguished between three sequential stages: (1) prevalence, (2) diagnosis and (3) HbA1c control. The prevalence of diabetes was assessed based on three parameters.

##### Prevalence

A dummy variable was used to indicate the prevalence of diabetes, taking the value of 1 for individuals who met at least one of the following criteria: (a) they answered ‘diabetes’ or ‘high blood sugar’ when asked in Wave 6 or previous waves, ‘Has a doctor ever told you that you had or currently have any of the conditions on this card?‘; (b) they answered ‘medication for diabetes’ to the question ‘Do you currently take medication at least once a week for the problems mentioned on this card?‘; or (c) they have an HbA1c level higher than 6.5%, but have not been diagnosed with diabetes or prescribed medication for it. This cutoff point for HbA1c levels is based on recommendations from the WHO [36] and the American Diabetes Association [37].

##### Diagnosis

The group of individuals identified as the prevalence group was used as the baseline for the next stage in the cascade; receiving a diagnosis. This variable categorised the prevalence group into two sub-groups: individuals with an HbA1c level above 6.5% who did not report a diabetes diagnosis or take diabetes medication (assigned a value of 0), and individuals who reported a diabetes diagnosis and/or take medication for it (assigned a value of 1).

##### Control

Individuals with a diabetes diagnosis were then divided into two groups: a controlled group and an uncontrolled group. The controlled group consisted of individuals with an HbA1c level below 7.0%. Although the cutoff for diagnosing diabetes was an HbA1c level of 6.5% in this study, the target for managing diabetes was set at a level of 7.0% [37].

#### Social capital variables

Arezzo and Giudici [38] previously operationalised the concepts of bonding and bridging social capital in relation to self-rated health, drawing on data from SHARE. In this previous article, the bonding social capital concept was operationalised through three variables: the number of family members in the social network, the frequency of family contact, and household size. The family contact variable was not included in this study because it overlaps with the number of family members, given that the number of family members influences the level of family contact. Additionally, we included a variable for having a partner in the household, as previous research has highlighted its importance in diabetes care [39–41]. To overcome multicollinearity, the household size variable excluded the partner.

Famnet, provided by SHARE, is based on a social network questionnaire and represents the number of family members identified by respondents in their social network. A value of 0 indicated no family members, while the maximum value was 7. The second variable, household size (excluding a partner), reflected the number of household members and ranged from 1 to 9. The third variable indicated the presence of a partner in the household.

Arezzo and Giudici [38] operationalised bridging social capital by three variables: the frequency of undertaking voluntary or charitable work, attending training or educational programmes, and participating in social or sports clubs. These variables are derived from two questions in the SHARE survey. The primary question asked was, ‘Which of the activities listed on this card, if any, have you engaged in over the past twelve months?’ Respondents who answered ‘No’ were given a value of 0, while those who answered ‘Yes’ were given a score from 1 to 4 according to their response to a follow-up question about how often they participated (1 = less often; 2 = almost every month; 3 = almost every week; 4 = almost daily).

#### Covariates

Previous research has shown associations between sociodemographic characteristics and both diabetes [3, 42, 43] and social capital [44, 45]. Furthermore, overall health has also been linked to sociodemographic factors [46, 47] and social capital [29, 38, 48]. Consequently, this analysis included covariates for sociodemographic characteristics (income, age, gender, and education), and for health indicators (activities of daily living (ADL), body mass index (BMI), and European depression (Euro-D^2^) scale) [49, 50].

After calculating standardised income, household income adjusted for household size^3^, respondents within each country were ranked and divided into five equally sized income groups (quintiles) to improve comparability across countries. Each quintile represents 20% of the national income distribution, ranging from the lowest (Q1) to the highest (Q5) quintile. In addition, both age and the square of age were included as continuous variables. Gender was treated as a binary variable, where a value of 1 indicated men and women served as the reference category. Education was categorised into three levels according to the International Standard Classification of Education 1997 (ISCED 97). In this dataset, ISCED 0–2 correspond to educational level 1, ISCED 3–4 to level 2, and ISCED 5–6 to level 3.

### Statistical analysis

We adopted a cascade of care (CoC) approach to examine the relationship between social capital and the various stages of diabetes care: prevalence, diagnosis and control. The analyses for each stage of the CoC were carried out using Stata (version 18.5), applying a conditional denominator approach. For each stage of the CoC, only participants who met the criteria of the preceding stage were included in the analysis for the next stage. For instance, the diagnosis stage considered only those identified with diabetes in the prevalence stage, while the control stage included only those who had been diagnosed with diabetes. Consequently, the associations with three outcome variables were studied: (1)prevalence of diabetes (total sample: all respondents); (2)diagnosis among those with diabetes (diabetes sample); and (3)control among those diagnosed (diabetes diagnosis sample). Therefore, the sample size exhibited variation at each stage, corresponding to the specific group under consideration.

A two-level logistic regression framework was applied to account for country clustering. Intraclass correlation coefficients (ICCs) were inspected to assess the multilevel structure. Wald tests examined the statistical significance of individual regression coefficients. Model fit was evaluated by comparing the Akaike Information Criterion (AIC) and Bayesian Information Criterion (BIC). In addition, stepwise models were constructed for each of the three CoC stages to identify mediating effects. Step 1 included only social capital variables, Step 2 added sociodemographic covariates (income quintiles, age, age², gender, and education), and Step 3 further incorporated health-related variables (ADL, BMI, and Euro-D Scale). By examining shifts in coefficient size and statistical significance across models, we examined how sociodemographic and health indicators confounded the relationship between social capital and each stage of CoC.

Any missing data was addressed through listwise deletion. This method facilitated the comparison of the same groups at different stages of the stepwise regression analysis. In addition, SHARE provided calibrated, cross-sectional, individual weights to correct for selection effects in analyses based on DBS data [33]. Furthermore, robustness checks were performed on the full dataset using multiple imputation to handle missing values.

## Results

### Descriptives

**Fig. 1:**
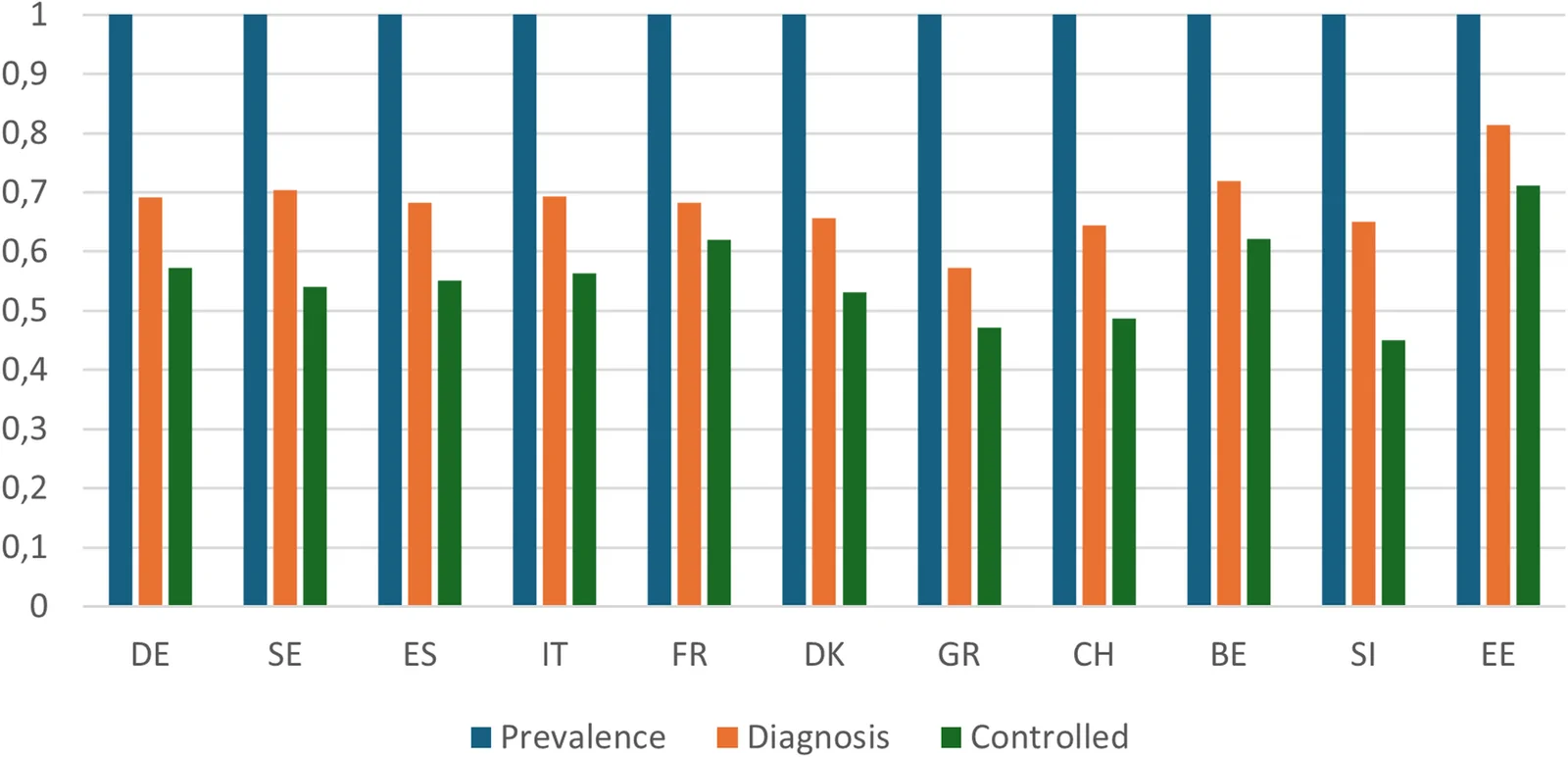
Diabetes care cascade by country DE = Germany, SE = Sweden, ES = Spain, IT = Italy, FR = France, DK = Denmark, GR = Greece, CH = Switzerland, BE = Belgium, SI = Slovenia, EE = Estonia

Figure 1 illustrates the diabetes CoC. Each bar represents the proportion of individuals who have successfully progressed through each stage of the CoC in the eleven European countries included in the study. The first bar is set to 100% and denotes the total number of diabetes cases. The second bar shows the proportion of diagnosed diabetes cases relative to the total diabetes cases. The final bar illustrates the proportion of people with diabetes whose condition is controlled, as measured by their HbA1c levels.

In this study, diabetes was prevalent in 3 773 individuals, accounting for 22.20% of the 16 993 individuals surveyed in total (95% CI:21.6%-22.8%). Of these individuals, 2 655 had received a diagnosis (70.37% of the respondents with diabetes (95% CI:68.9%-71.8%)). Therefore, 29.63% (*n* = 1 118) remained undiagnosed. Of the diagnosed diabetes cases, 81.69% (2 169 out of 2 655) had HbA1c levels below 7%, indicating effective control of their condition (95% CI:80.2%-83.2%). Table 1 presents the descriptive characteristics related to the total sample, diabetes sample and the diabetes diagnosis sample.

**Table 1:** Descriptive characteristics by sample

	Total sample		Diabetes sample		Diabetes diagnosis sample	
	Absolute numbers	Percentage/S.D.	Absolute numbers	Percentage/S.D.	Absolute numbers	Percentage/S.D.
Gender						
Men	7353	43.27%	1769	46.89%	1283	48.32%
Women	9640	56.73%	2004	53.11%	1372	51.68%
Average Age	67	S.D.=9.29	69.26	S.D.=9.08	69.89	S.D.=8.89
Education						
Low	5429	31.95%	1514	40.13%	1082	40.75%
Medium	6966	40.99%	1498	39.70%	1060	39.92%
High	4598	27.06%	761	20.17%	513	19.32%
Income						
Quintile1	3333	19.61%	901	23.88%	669	25.20%
Quintile2	3628	21.35%	930	24.65%	673	25.35%
Quintile3	3557	20.93%	788	20.89%	551	20.75%
Quintile4	3440	20.24%	645	17.10%	431	16.23%
Quintile5	3035	17.86%	509	13.49%	331	12.47%
Average BMI	26.37	S.D.=4.70	28.90	S.D.=5.23	29.55	S.D.=5.37
Average ADL Score	0.17	S.D.=0.64	0.28	S.D.=0.81	0.34	S.D.=0.88
Average Euro-D scale	2.34	S.D.=2.11	2.65	S.D.=2.28	2.86	S.D.=2.34
Frequency Voluntary						
Not	13,544	79.70%	3140	83.22%	2249	84.71%
Less often	668	3.93%	134	3.55%	86	3.24%
Almost every month	985	5.80%	193	5.12%	118	4.44%
Almost every week	1402	8.25%	239	6.33%	160	6.03%
Almost every day	394	2.32%	67	1.78%	42	1.58%
Frequency Programme						
Not	14,462	85.11%	3402	90.17%	2413	90.89%
Less often	1429	8.41%	218	5.78%	147	5.54%
Almost every month	481	2.83%	59	1.56%	42	1.58%
Almost every week	579	3.41%	88	2.33%	49	1.85%
Almost every day	42	0.25%	6	0.16%	4	0.15%
Frequency Club						
Not	11,058	65.07%	2748	72.83%	1971	74.24%
Less often	513	3.02%	109	2.89%	77	2.90%
Almost every month	987	5.81%	217	5.75%	149	5.61%
Almost every week	3617	21.29%	565	14.97%	365	13.75%
Almost every day	818	4.81%	134	3.55%	93	3.50%
Average #FamilyMembers	2.15	S.D.=1.32	2.13	S.D.=1.30	2.09	S.D.=1.28
Partner						
Yes	12,521	73.68%	2699	71.53%	1864	70.21%
No	4472	26.32%	1074	28.47%	791	29.79%
Average Household size	1.31	S.D.=0.70	1.31	S.D.=0.71	1.31	S.D.=0.71
* Sample size*	16,993		3773		2655	

*S.D.* standard deviation

Based on Table 2, the prevalence of diabetes varied among the countries included in the sample. The greatest prevalence was observed in Greece (38.98%) and Spain (37.23%). In contrast, the lowest numbers were reported in Denmark (12.83%), Switzerland (15.87%), Belgium (19.73%), and Sweden (19.85%). Figure 1 shows that Estonia had the highest percentage of people receiving a diabetes diagnosis of the prevalent cases (81.44%), followed by Belgium (71.88%) and Sweden (70.45%). The lowest numbers were found in Greece (57.25%), Switzerland (64.35%), and Slovenia (64.96%). When we analysed the proportion of the diagnosed diabetes cases who had effectively controlled their diabetes (Table 2)^4^, France^5^ emerged as the frontrunner, with 90.70% of diabetes cases successfully controlling their condition. Estonia and Belgium followed behind, with 87.34% and 86.34%. By contrast, only 69.30% of individuals with a diabetes diagnosis in Slovenia successfully had managed their diabetes. Switzerland and Sweden also had comparatively lower numbers, of 75.54% and 76.78%.

**Table 2:**
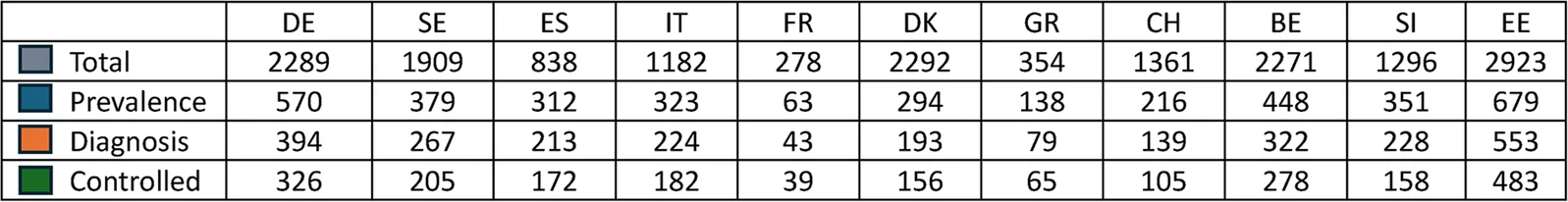
Absolute numbers of the cascade stages by country

DE = Germany, SE = Sweden, ES = Spain, IT = Italy, FR = France, DK = Denmark, GR = Greece, CH = Switzerland, BE = Belgium, SI = Slovenia, EE = Estonia

### General model fit

Examining the results of the stepwise and multilevel approaches in Tables 3, 4 and 5 shows that AIC and BIC consistently decreased with the inclusion of additional covariates. This suggests improved model fit at each step of the Diabetes CoC and indicates that models incorporating sociodemographic characteristics and health indicators consistently provided the best overall fit among the models considered. The random intercept variance at the country level revealed modest heterogeneity in the prevalence and control stages between countries, with variances ranging from 0.12 to 0.16 and ICC values of 3–5%. This suggests that a small proportion of outcome variation was due to differences between countries. In contrast, diagnosis exhibited much lower country variance (0.04–0.06) and ICC values of 1–2%, suggesting that diagnosis rates among individuals with diabetes were more consistent across countries.

**Table 3:** CoC: prevalence stage

Stepwise multilevel logistic regression						
Prevalence	**Step 1**		**Step 2**		**Step 3**	
	*Logit coefficients*	*S.D.*	*Logit coefficients*	*S.D.*	*Logit coefficients*	*S.D.*
Constant	-0.8587***	0.1303	-6.0429***	1.3634	-7.3961***	1.5395
Bonding social capital						
Partner	-0.1099	0.0710	-0.0169	0.0856	-0.0208	0.0805
Household size	-0.0581	0.0356	0.0295	0.0250	0.0072	0.0226
#FamilyMembers	-0.0243	0.0181	-0.0149	0.0172	-0.0164	0.0210
Bridging social capital						
Frequency Voluntary	-0.0189	0.0204	-0.0127	0.0214	-0.0051	0.0230
Frequency Programme	-0.2137***	0.0426	-0.1019*	0.0394	-0.0946*	0.0432
Frequency Club	-0.0965***	0.0149	-0.0753***	0.0133	-0.0519**	0.0179
Sociodemographic						
Age			0.1342***	0.0379	0.0877*	0.0416
Age²			-0.0009**	0.0003	-0.0005	0.0003
Men			0.2378*	0.0956	0.2508***	0.0684
Medium education			-0.1154	0.0644	-0.0631	0.0736
High education			-0.3138***	0.0638	-0.1915**	0.0662
Quintile2			-0.0715	0.0623	-0.0595	0.0675
Quintile3			-0.1525**	0.0543	-0.0963	0.0571
Quintile4			-0.3381***	0.0460	-0.2442***	0.0573
Quintile5			-0.3275***	0.0767	-0.2479**	0.0906
Health						
ADL					0.1077**	0.0328
BMI					0.0960***	0.0069
Euro-D					0.0332***	0.0090
Country var(_cons)	0.1450	0.0552	0.1240	0.0464	0.1216	0.0448
ICC	0.0422	0.0154	0.0363	0.0131	0.0356	0.0127
Model fit	AIC = 17747.03	BIC = 17808.96	AIC = 17500.13	BIC = 17577.54	AIC = 16849.86	BIC = 16927.27

*p* < 0.001***, *p* < 0.01**, *p* < 0.05*

*S.D.* standard deviation, *ICC* Intraclass Correlation Coefficient

**Table 4:** CoC: Diagnosis stage

Stepwise multilevel logistic regression						
Diagnosis	Step 1		Step 2		Step 3	
	*Logit coefficients*	*S.D.*	*Logit coefficients*	*S.D.*	*Logit coefficients*	*S.D.*
Constant	1.1680***	0.0835	-8.1619***	1.9003	-10.5672	2.085
Bonding social capital						
Partner	-0.1632*	0.0640	-0.0726	0.0859	-0.0345	0.0893
Household size	-0.0201	0.0517	0.0979	0.0608	0.0700	0.0695
#FamilyMembers	-0.0945	0.0770	-0.0956	0.0767	-0.0917	0.0678
Bridging social capital						
Frequency Voluntary	-0.0661	0.0426	-0.0693	0.0447	-0.0560	0.0449
Frequency Programme	-0.1466**	0.0523	-0.0681	0.0567	-0.0451	0.0592
Frequency Club	-0.0627	0.0434	-0.0621	0.0413	-0.0359	0.0444
Sociodemographic						
Age			0.2442***	0.0558	0.2225***	0.0564
Age²			-0.0016***	0.0004	-0.0014**	0.0004
Men			0.2000	0.1380	0.3225*	0.1349
Medium education			0.1260	0.1076	0.1784	0.0986
High education			0.0938	0.1275	0.2399	0.1429
Quintile2			-0.1322	0.0964	-0.1507	0.1026
Quintile3			-0.2999*	0.1204	-0.2147*	0.1082
Quintile4			-0.2525	0.1377	-0.1513	0.1516
Quintile5			-0.3113*	0.1271	-0.2328	0.1313
Health						
ADL					0.2004***	0.0386
BMI					0.0884***	0.0163
Euro-D					0.1289***	0.0332
Country var(_cons)	0.0620	0.0359	0.0639	0.0325	0.0431	0.0211
ICC	0.0185	0.0105	0.0191	0.0095	0.0129	0.0063
Model fit	AIC = 4626.52	BIC = 4676.41	AIC = 4552.11	BIC = 4614.47	AIC = 4332.59	BIC = 4394.95

*p* < 0.001***, *p* < 0.01**, *p* < 0.05*

*S.D.* standard deviation, *ICC* Intraclass Correlation Coefficient

**Table 5:** CoC: Control stage

Stepwise multilevel logistic regression						
Controlled	Step 1		Step 2		Step 3	
	*Logit coefficients*	*S.D.*	*Logit coefficients*	*S.D.*	*Logit coefficients*	*S.D.*
Constant	1.3170***	0.1664	3.3032	3.4798	3.5786	3.7265
Bonding social capital						
Partner	0.2605**	0.0845	0.3073**	0.1084	0.2988**	0.1104
Household size	-0.0337	0.0894	-0.0261	0.0883	-0.0257	0.0886
#FamilyMembers	0.0026	0.0548	-0.0152	0.0607	-0.0085	0.0631
Bridging social capital						
Frequency Voluntary	-0.0018	0.0880	-0.0032	0.0885	-0.0139	0.0900
Frequency Programme	-0.0274	0.1446	-0.0419	0.1234	-0.0429	0.1103
Frequency Club	0.0751	0.0608	0.0760	0.0601	0.0749	0.0622
Sociodemographic						
Age			-0.0625	0.0965	-0.0371	0.0966
Age²			0.0005	0.0007	0.0003	0.0006
Men			-0.2950*	0.1301	-0.2964*	0.1271
Medium education			0.1912	0.1882	0.1890	0.1933
High education			0.2412	0.3092	0.2079	0.3079
Quintile2			0.1329	0.2368	0.1286	0.2378
Quintile3			0.3113	0.1947	0.2951	0.1864
Quintile4			-0.0629	0.1288	-0.1092	0.1304
Quintile5			0.0411	0.1125	0.0132	0.1042
Health						
ADL					-0.0346	0 0.0568
BMI					-0.0350*	0.0139
Euro-D					0.0162	0.0322
Country var(_cons)	0.1456	0.0525	0.1522	0.0566	0.1602	0.0619
ICC	0.0424	0.0146	0.0442	0.0157	0.0464	0.0171
Model fit	AIC = 2485.92	BIC = 2532.99	AIC = 2473.65	BIC = 2532.49	AIC = 2459.97	BIC = 2518.82

*p* < 0.001***, *p* < 0.01**, *p* < 0.05*

*S.D.* standard deviation, *ICC* Intraclass Correlation Coefficient

### Determinants of CoC

#### Prevalence

Table 3 presents the results of a multilevel logistic regression model constructed using a stepwise approach, with diabetes prevalence as the dependent variable. The table shows an association between bridging social capital and diabetes prevalence, indicating that individuals who participated more in training or educational programmes and in social or sports clubs tended to have lower diabetes prevalence. While this correlation weakened after adding the sociodemographic variables and health indicators, it remained significant. The attenuation of the bridging social capital variables suggested that the relation between social capital and diabetes prevalence was partially mediated by the included sociodemographic factors and health indicators. Additional descriptive analyses confirm this trend (Appendix: section A, Tables 6, 7 and 8), indicating that non-participants tended to be older than participants. Poorer health, lower levels of education, and lower income were also associated with reduced participation in programmes and club activities.

Age, gender, education, and income all had a significant relationship with diabetes prevalence. Specifically, older adults, men, individuals with lower education levels compared to those with higher education, and those with lower income compared to individuals with higher income, were more likely to have diabetes. The significance of the age-squared variable indicated that diabetes prevalence initially increased with age, but the correlation diminished at older ages. After incorporating the health indicators, the parameters for age, income, and education all decreased in magnitude, but maintained their significant correlations (except for the age-squared parameter). Gender, however, showed a slight increase after accounting for health indicators. Regarding the health indicators, the parameters related to ADL, BMI, and the Euro-D Scale were all positively significant, indicating that poorer physical and mental health correlated with increased diabetes prevalence.

#### Diagnosis

The next stage in the diabetes CoC was receiving a diagnosis after the condition had developed (Table 4). All indicators of social capital showed a negative trend, suggesting that higher levels of social capital were associated with a lower likelihood of receiving a diabetes diagnosis. Participation in training or educational programmes, and having a partner in the household, were the only social capital variables that were significantly associated with a lower likelihood of receiving a diagnosis. Nevertheless, this significant association disappeared when sociodemographic variables and health indicators were included.

Sociodemographic factors revealed that age was a significant predictor: older individuals were more likely to receive a diagnosis. Incorporating a quadratic age term showed that this correlation diminished with increasing age. Higher income levels were associated with a lower probability of receiving a diagnosis. The association between gender and receiving a diagnosis became significant in step 3, with men being more likely to be diagnosed than women. Examining health indicators revealed that individuals in poorer health were more likely to be diagnosed with diabetes. The parameters related to ADL, BMI, and the Euro-D scale were all significantly positive.

The observed negative trend in social capital variables contradicted Hypotheses 1b and 2b, which predicted a positive correlation between bonding and bridging social capital and the likelihood of receiving a diagnosis. Instead, the strongest predictors of receiving a diagnosis were age, gender, and the health indicators. Additional descriptive analyses revealed a relationship between the social capital variables identified as significant in the first step (participation in programmes and having a partner in the household) and these key factors (Appendix, Section B, Tables 9, 10 and 11). This indicates an indirect effect. Among people with diabetes, men were more likely to have a partner than women. Individuals without a partner tended to be older. Higher levels of depressive symptoms were correlated with reduced participation in programmes. Furthermore, greater participation in programmes was associated with being younger and having a lower BMI.

#### Control

The final step in the diabetes CoC examined whether individuals diagnosed with diabetes had effectively controlled their condition, based on their HbA1c values (Table 5). When analysing social capital parameters, one factor stood out: having a partner in the household was positively associated with better diabetes control. This association became stronger when accounting for sociodemographic variables and health indicators. Men were associated with poorer HbA1c levels than women, with gender emerging as the only significant sociodemographic factor. Of the health indicators examined, only BMI was significant, with higher BMI being negatively associated with diabetes control.

#### Missing imputations

Multiple Imputation (MI) was used as a robustness check to address missing data in the three previously analysed samples. The MI sample sizes are now 18 916 for the total sample, 4 288 for the diabetes sample, and 3 037 for the diabetes diagnosis sample. Applying the same methodology, similar conclusions were obtained, despite some parameter fluctuations. When diabetes prevalence was the dependent variable, bridging social capital remained significantly associated, partly mediated by sociodemographic and health indicators. Unlike earlier results, having a partner in the household was also significantly correlated with diabetes prevalence in step 1, but this effect disappeared in later steps. In the analysis of diabetes diagnosis, partner status, participation in programmes, and in club activities were negatively linked to lower likelihoods of diagnosis. However, these significant associations did not persist in the final step. Overall, older ages (this relation weakens with age), men, and people in poorer health were more likely to receive a diabetes diagnosis. The control stage, with the smallest sample size, was most affected by additional respondents. In this stage, coefficients changed most compared to the listwise deletion results. Unlike previous results, BMI was the only significant predictor of diabetes control, with higher BMI associated with poorer HbA1c control.

## Discussion

This research examined social capital to address gaps in the diabetes CoC. The study analysed the relationship between bonding and bridging social capital and diabetes prevalence, diagnosis and control. Of a sample of 16 993 adults aged 50 and over from 11 European countries, 3 773 (22.20%) had diabetes. Of these, 2 655 (70.37%) were diagnosed. Among those who were diagnosed, 2 169 (81.69%) had HbA1c levels below 7%, indicating effective glycaemic control.

While bonding social capital was not associated with diabetes prevalence (hypothesis 1a rejected), bridging social capital was (hypothesis 2a accepted). Participation in training or educational programmes, and in social or sports clubs, was linked to reduced prevalence. This relationship was partly mediated by sociodemographic characteristics and health indicators. Prevalence was higher among the elderly, men, those from lower income groups, with less education, and in poorer health. Our findings are in line with other studies that have identified a relationship between the prevalence of diabetes and bridging social capital. For instance, Shibayama, Noguchi [24] found that participation in social activities made middle-aged Japanese adults less vulnerable to diabetes. Similarly, Hilding, Shen [23] reported that social participation reduced the risk of prediabetes in Swedish middle-aged men, and lowered the risk of type 2 diabetes in Swedish middle-aged women. Nevertheless, they also found that having a partner lowered men’s diabetes risk, a finding that was not supported by our research [23]. Nevertheless, comparison is difficult because they examined a different population (Swedish adults aged 35–56).

After the prevalence stage, the next stage in the CoC was diabetes diagnosis, which was primarily associated with age, gender, and health status: older adults, men, and those in poorer health were more likely to be diagnosed. While participation in training and educational programmes, and having a partner in the household, were initially significantly associated with a reduced likelihood of diagnosis. These associations vanished after adjusting for sociodemographic and health factors, indicating an indirect link between social capital and diagnosis (hypotheses 1b and 2b rejected). Lower levels of social capital were more prevalent among older adults and individuals in poorer health, both of whom were more likely to receive a diabetes diagnosis. This pattern may account for the negative association between social capital and receiving a diagnosis. Furthermore, individuals not traditionally considered at high risk for diabetes, such as women, younger adults, and those with higher incomes, are diagnosed less often, highlighting the need for broader screening and prevention strategies to encompass populations beyond those traditionally considered high risk. Supporting this, Kautzky-Willer, Leutner [51] found that, at the time of type 2 diabetes diagnosis, women exhibit a greater burden of risk factors than men, including excess weight, obesity, and hypertension, a disparity that may indicate delayed detection in women. Lee, Armstrong [52] reported that younger adults were more likely to remain undiagnosed than older adults. The atypical finding regarding higher-income groups may reflect selection bias, as noted in the limitations.

After the diagnosis stage, the next stage was diabetes control; being a man and having a high BMI were linked to poorer HbA1c levels. Although bridging social capital variables were not associated with diabetes control (hypothesis 2c rejected), one bonding social capital parameter, having a partner in the household, was significantly associated with improved HbA1c levels, even after controlling for sociodemographic and health indicators (hypothesis 1c accepted). These findings were consistent with previous research, which showed better diabetes management in individuals with a partner [39, 40]. Diabetes control typically requires adopting lifestyle changes, including consistent medication use and healthy dietary habits [1]. Maintaining these routines requires motivation and self-discipline, which can be challenging without support. Having a partner can enhance social capital by offering emotional encouragement, practical assistance, and reinforcement of treatment adherence [39, 40]. The present study found no correlation between participation and diabetes control. However, previous studies indicated that increased civic engagement is associated with improved glycaemic control [28], and that community participation enhances self-efficacy in women with gestational diabetes mellitus [53]. Notably, comparing results across studies is complex due to variations in geography and the present study’s focus on adults aged 50+, a group that generally reported lower participation than the broader population.

Although this study offers valuable insights, several limitations should be acknowledged. First, its cross-sectional design limits causal inference and raises the possibility of reverse causality. Individuals who are less healthy may invest less time building and maintaining their social networks. Second, a selection effect arises in later stages of the CoC, where only respondents with diabetes or a diabetes diagnosis are selected. This may account for unexpected findings, like the negative association between certain social capital variables and diabetes care at the diagnosis and control stages. Notably, these negative effects are generally not statistically significant or disappear after controlling for sociodemographic and health factors. Third, the sampling method may introduce bias, as individuals who are not picked up by the healthcare system are likely also challenging for this study to access.

Overall, the results suggested that gaps in diabetes care were not random but were partly related to levels of social capital. Having more bridging social capital was linked to lower diabetes prevalence, while having a partner was found to be associated with better diabetes control, and an indirect association was observed at the diagnosis stage. Therefore, social capital appeared to be intertwined with multiple aspects of diabetes management. Navigating this condition effectively requires more than medical treatment; social relationships and participation have a contributory role within the broader context of diabetes care [14, 15, 17, 23, 28, 40]. Building robust community networks is essential for improving health outcomes. European initiatives such as Feel4Diabetes, a four-year project that encourages families to prevent diabetes [54], and the global Cities for Better Health partnership, which operates in 27 European cities to address barriers to healthy living, demonstrate how community involvement can support prevention [55]. These projects and the current research both emphasise that community participation and social ties build supportive networks for effective diabetes care. Future research should utilise longitudinal data to more clearly elucidate the causal pathways by which social capital influences diabetes care. By tracking individuals across different stages of the care continuum, researchers can more effectively clarify the potential selection effects identified in this study.

## Conclusion

This study underscores the multifaceted role of social capital in diabetes care for older adults in Europe. Bridging social capital was linked to a lower prevalence of diabetes, while having a partner appeared to support better glycaemic control. In addition, social capital was indirectly related to the likelihood of receiving a diagnosis. Together, these findings point to the combined importance of a medical and social approach in improving diabetes outcomes. Efforts to strengthen community engagement, involve family members, and extend prevention initiatives to groups not usually seen as high risk may help to reduce existing gaps in care. Longitudinal studies are needed to better understand the direction of these relationships, and to guide interventions that bring together clinical and social approaches to diabetes management.

## Data Availability

The data that support the findings of this study come from the Survey of Health, Ageing, and Retirement in Europe (SHARE), Wave 6, including the Dried Blood Spot (DBS) data. These data are available upon registration and data access approval from the SHARE Research Data centre. The SHARE data are not publicly available due to ethical and legal restrictions but can be obtained free of charge for scientific use from the SHARE website (https://share-project.org) after submitting a data access application.

## Appendix

### Section A: additional descriptive analysis of the total sample

**Table 6:** Mean age and health indicators by frequency of participation

	Means by participation frequency (s.e.)			
Freq.Programme	Age	BMI	ADL	Euro-D
0	68.37 (0.08)	27.15 (0.04)	0.20 (0.01)	2.42 (0.02)
1	61.34 (0.18)	26.37 (0.12)	0.06 (0.01)	1.88 (0.05)
2	62.19 (0.35)	26.37 (0.21)	0.06 (0.01)	1.92 (0.08)
3	65.93 (0.32)	25.96 (0.17)	0.07 (0.02)	2.03 (0.08)
4	62.33 (1.28)	26.05 (0.60)	0.17 (0.11)	1.95 (0.28)

Freq.Club	**Age**	**BMI**	**ADL**	**Euro-D**
0	68.20 (0.09)	27.35 (0.05)	0.22 (0.01)	2.59 (0.02)
1	65.88 (0.40)	27.29 (0.21)	0.16 (0.03)	2.01 (0.08)
2	66.87 (0.29)	27.08 (0.15)	0.11 (0.01)	2.00 (0.06)
3	66.20 (0.14)	26.26 (0.07)	0.09 (0.01)	1.85 (0.03)
4	65.85 (0.30)	25.73 (0.13)	0.07 (0.01)	1.77 (0.06)

**Table 7:** Cross-table of education level and frequency of participation

	Education level					
Freq.Programme	Low		Medium		High	
0	5148	(94.82%)	6026	(86.51%)	3288	(71.51%)
1	137	(2.52%)	597	(8.57%)	695	(15.12%)
2	43	(0.79%)	149	(2.14%)	289	(6.29%)
3	96	(1.77%)	177	(2.54%)	306	(6.66%)
4	5	(0.09%)	17	(0.24%)	20	(0.43%)
Total	5429	(100%)	6966	(100%)	4598	(100%)

Freq.Club	**Low**		**Medium**		**High**	
0	4249	(78.26%)	4500	(64.60%)	2309	(50.22%)
1	119	(2.19%)	210	(3.01%)	184	(4.00%)
2	195	(3.59%)	446	(6.40%)	346	(7.53%)
3	715	(13.17%)	1506	(21.62%)	1396	(30.36%)
4	151	(2.78%)	304	(4.36%)	363	(7.89%)
Total	5429	(100%)	6966	(100%)	4598	(100%)

**Table 8:** Cross-table of income quintile and frequency of participation

	Income Quintile									
Freq.Programme	Q1		Q2		Q3		Q4		Q5	
0	3111	(93.34%)	3271	(90.16%)	3102	(87.21%)	2734	(79.48%)	2244	(73.94%)
1	103	(3.09%)	186	(5.13%)	268	(7.53%)	419	(12.18%)	453	(14.93%)
2	40	(1.20%)	67	(1.85%)	72	(2.02%)	143	(4.16%)	159	(5.24%)
3	70	(2.10%)	101	(2.78%)	107	(3.01%)	137	(3.98%)	164	(5.40%)
4	9	(0.27%)	3	(0.08%)	8	(0.22%)	7	(0.20%)	15	(0.49%)
Total	3333	(100%)	3628	(100%)	3557	(100%)	3440	(100%)	3035	(100%)

Freq.Club	**Q1**		**Q2**		**Q3**		**Q4**		**Q5**	
0	2462	(73.87%)	2497	(68.83%)	2353	(66.15%)	2064	(60.00%)	1682	(55.42%)
1	82	(2.46%)	90	(2.48%)	117	(3.29%)	112	(3.26%)	112	(3.69%)
2	141	(4.23%)	190	(5.24%)	195	(5.48%)	244	(7.09%)	217	(7.15%)
3	561	(16.83%)	694	(19.13%)	727	(20.44%)	824	(23.95%)	811	(26.72%)
4	87	(2.61%)	157	(4.33%)	165	(4.64%)	196	(5.70%)	213	(7.02%)
Total	3333	(100%)	3628	(100%)	3557	(100%)	3440	(100%)	3035	(100%)

### Section B: additional descriptive analysis of the diabetes sample

**Table 9:** Mean age and health indicators by frequency of participation

	Means by participation frequency (s.e.)		
Freq.Programme	Age	BMI	Euro-D
0	69.83 (0.16)	28.95 (0.09)	2.72 (0.04)
1	62.34 (0.49)	28.63 (0.35)	2.19 (0.13)
2	64.53 (0.94)	29.33 (0.72)	2.03 (0.23)
3	68.22 (0.83)	27.78 (0.47)	1.84 (0.20)
4	62.83 (4.04)	27.05 (1.38)	1.00 (0.63)

**Table 10:** Mean age and Euro-D scale by partner categories

	Means by partner variable categories (s.e.)	
Partner	Age	Euro-D
No	72.63 (0.30)	3.17 (0.07)
Yes	67.93 (0.16)	2.45 (0.04)

**Table 11:** Cross-table of partner and gender

	Gender			
Partner	Women		Men	
No	748	(37.33%)	326	(18.43%)
Yes	1256	(62.67%)	1443	(81.57%)
Total	2004	(100%)	1769	(100%)

## Footnotes

1 HbA1c measures the average blood glucose concentration over a period of 90 days preceding a blood test.

2 The Euro-D scale is a questionnaire designed to identify depressive symptoms in older adults in Europe [50].

3 Derived from: Standardised income= Household income/(Household size^0.36).

4 This contrasts with Fig. 1, which presents the proportion of controlled cases among all diabetes cases (both diagnosed and undiagnosed). Meanwhile, the following numbers represent the percentage of controlled cases among diagnosed diabetes cases only, based on Table 2.

5 The number of respondents from France was limited as eligibility was restricted to four districts for logistical reasons.
